# A feasibility study of a handmade ultrasound-guided phantom for paracentesis

**DOI:** 10.1186/s12909-024-05339-9

**Published:** 2024-03-29

**Authors:** Chien-Tai Huang, Chih-Hsien Lin, Shao-Yung Lin, Sih‑Shiang Huang, Wan-Ching Lien

**Affiliations:** 1 Department of Emergency Medicine, National Taiwan University Hospital, Hsin-Chu Branch, Hsin-Chu, Taiwan; 2 Department of Emergency Medicine, National Taiwan University Hospital, No.7, Chung-Shan South Road, Taipei, 100 Taiwan; 3 Department of Emergency Medicine, College of Medicine, National Taiwan University, Taipei, Taiwan

**Keywords:** Phantom, Ultrasound, Procedure, Paracentesis

## Abstract

**Background:**

Simulation-based training is effective for ultrasound (US)-guided procedures. However, commercially developed simulators are costly. This study aims to evaluate the feasibility of a hand-made phantom for US-guided paracentesis.

**Methods:**

We described the recipe to prepare an agar phantom. We collected the US performance data of 50 novices, including 22 postgraduate-year (PGY) residents and 28 undergraduate-year (UGY) students, who used the phantom for training, as well as 12 emergency residents with prior US-guided experience. We obtained the feedback after using the phantom with the Likert 5-point scale. The data were presented with medians and interquartile ranges (IQRs) and analyzed by the Wilcoxon rank sum test.

**Results:**

While emergency residents demonstrated superior performance compared to trainees, all trainees exhibited acceptable proficiency (global rating of ≥ 3, 50/50 vs. 12/12, *p* = 1.000) and comparable needle steadiness [5 (5) vs. 5 (5), *p* = 0.223]. No significant difference in performance was observed between PGYs [5 (4–5)] and UGYs [5 (4–5), *p* = 0.825]. No significant differences were observed in terms of image stimulation, puncture texture, needle visualization, drainage simulation, and endurance of the phantom between emergency residents and trainees. However, experienced residents rated puncture texture and draining fluid as “neutral” (3/5 on the Likert scale). The cost of the paracentesis phantom is US$16.00 for at least 30 simulations, reducing it to US$6.00 without a container.

**Conclusions:**

The paracentesis phantom proves to be a practical and cost-effective training tool. It enables novices to acquire paracentesis skills, enhances their US proficiency, and boosts their confidence. Nevertheless, further investigation is needed to assess its long-term impact on clinical performance in real patients.

**Trial registration:**

NCT04792203 at the ClinicalTrials.gov.

**Supplementary Information:**

The online version contains supplementary material available at 10.1186/s12909-024-05339-9.

## Introduction

Clinical procedures involve a complex combination of technical skills and cognitive decision-making. Achieving expert performance and sustaining skills necessitate deliberate practice [1]. Traditionally, procedural skills were acquired, and experience accumulated through direct application on real patients. However, concerns about patient safety and rights have escalated with inexperienced physicians performing procedures directly on patients. Simulation-based medical education provides an alternative for skill proficiency [2], particularly in ultrasound (US)-guided procedures [3].

Paracentesis is a commonly encountered procedure in clinical practice. The use of ultrasound guidance diminishes the risk of a dry tap (failure to obtain fluid) during paracentesis and reduces the likelihood of complications such as bleeding, abdominal hematoma, and puncture site infection [4, 5]. Additionally, US-guided procedures are integral to emergency medicine training [6]. However, commercially developed simulators for US-guided procedures are often prohibitively expensive for many emergency departments.

An increasing number of low-cost, handmade phantoms have been developed for US-guided biopsy, thoracocentesis, and pericardiocentesis [3, 7–17]. However, options for paracentesis remain limited [18–20]. Furthermore, more evidence is needed to assess the learning impact of using handmade phantoms for paracentesis training. This study aims to evaluate the feasibility of a handmade phantom for US-guided paracentesis.

## Methods

This prospective study was conducted at the Emergency Department of the National Taiwan University Hospital (NTUH) from August 2022 to July 2023. It was approved by the institutional review board of the NTUH (202011111RIND) and registered at ClinicalTrials.gov (NCT04792203). Informed consent was obtained from each participant.

### Phantom preparation

1. Agar substrate

The agar substrate was created by dissolving 10 g of agar powder in 1000 cc of water. After thorough heating to melt the agar powder, the solution underwent filtration to remove impurities. The resulting clear solution was tinted with dark blue food coloring additives.

2. Paracentesis phantom

**Fig. 1:**
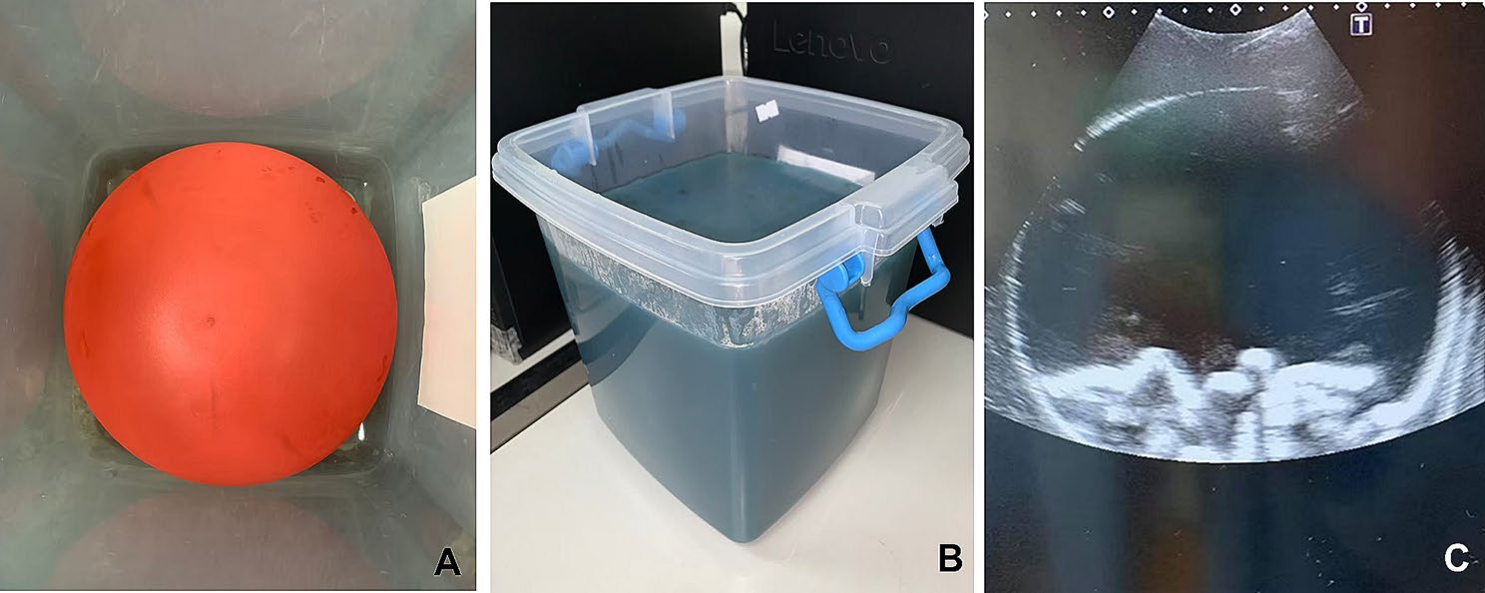
(**A**) The balloon was tied in the container; (**B**) The phantom; (**C**) The simulated sonographic image of the phantom

A cotton rope, at least 30 cm in length, was inserted into a 10-inch balloon to simulate the bowel. The balloon was then filled with yellowish water dyed with food coloring additives to mimic ascites. After tying the balloon securely, it was affixed to the bottom of the container using super glue (Fig. 1A). The balloon was covered with the agar substrate, replicating the appearance of human skin and the subcutaneous area. The thickness of the covering could be adjusted based on different body habitus. The phantom was refrigerated for a minimum of 4 h to enhance its longevity (Fig. 1B).

The resulting phantom exhibited easily distinguishable echogenic structures (Fig. 1C, Supplementary Video). The balloon effectively delineated boundaries between the peritoneum and the subcutaneous area.

### US-guided paracentesis using the hand-made phantom

We recruited 50 trainees, comprising 22 postgraduate-year 1 (PGY-1) residents and 28 undergraduate-year (UGY) students, for participation in a US training curriculum. To assess their experience and confidence in using US, the trainees completed a survey using a 5-point Likert scale (1 = not confident at all; 2 = slightly confident; 3 = somewhat confident; 4 = fairly confident; 5 = completely confident). Subsequently, they attended a 30-minute didactic session covering the theory of US and US-guided paracentesis, followed by small-group hands-on training utilizing the agar phantom. The instructors, were certified by the Taiwan Society of Emergency Medicine.

Following the curriculum, all trainees underwent a skill test, performing paracentesis. The performance was evaluated using an assessment form (Table 1) in which the items of the assessment was developed to to encompass the training domains based on expert consensus. Three experts, certified by the Taiwan Society of Emergency Medicine and with over 10 years of US experience, participated in establishing this consensus.

**Table 1:** The checklist trainees’ performance for paracentesis

Item	1	2	3	4	5
Ultrasound-guided localization	Many unnecessary probe/needle movements		Efficient time/motion but some unnecessary probe/needle movements		Efficient probe/needle movements
Visualization of needle	Needle tip not seen during entering and difficult to locate after entry		Needle tip not seen during entering but easily and quickly located after entry		Tip clearly seen during entering
Fluid aspiration	No fluid is aspirated		Fluid is aspirated		Fluid is aspirated smoothly
Needle steadiness during aspiration	Many unnecessary movements; needle pulled out of the cavity or touched structures		Some unnecessary needle movements		Minimal needle movement during aspiration
Global scores	Unacceptable performance; multiple major inadequacies	Unacceptable performance; some major inadequacies	Acceptable performance; minor inadequacies	Acceptable performance	Exceptional performance; expert provider

Two independent evaluators, not involved in enrollment and training, graded the performance—one on-site, and the other assessed video recordings with trainee faces masked. Subsequently, trainees provided feedback on the phantom through a survey using a 5-point Likert scale (Supplementary Table 1).

Additionally, 12 emergency residents were enrolled to use the phantom without didactics and hands-on training. Their performance was graded, and a survey regarding the phantom was collected.

US machines (Xario 100, Canon, Japan, and Arietta 780, Fujifilm Healthcare, Japan) equipped with a 2–5 MHz curvilinear transducer were used.

### Statistical analysis

All data were analyzed by SAS software (SAS 9.4, Cary, North Carolina, USA). Initially, we conducted the Shapiro-Wilk test to assess the normality of continuous data. If the data did not follow a normal distribution, it was presented using medians and interquartile ranges (IQRs). For the comparison between residents and trainees, as well as between PGYs and UGYs, we employed Wilcoxon’s rank-sum test.

To assess inter-rater reliability between two evaluators for the items on the assessment form and global scores, we utilized the intraclass correlation coefficient (ICC) with 95% confidence intervals (CIs). The Spearman correlation coefficient was used to evaluate the relationship between the total score and the global score. The total score represented the sum of each item on the assessment form. The internal reliability of the assessment form was estimated by employing Cronbach’s alpha coefficient [21]. A p-value less than 0.05 was considered statistically significant.

## Results

Following the assessment of normality, it was determined that the scores for each item on the assessment form, the global score, and feedback to the phantom were not normally distributed (all *p* < 0.0001). Therefore, these data were reported using medians and IQRs.

### US performance

The 50 trainees were all considered US novices (Table 2). The 12 emergency residents had previous experience with US-guided paracentesis on more than 20 real patients. The ICC for the global score was 0.94 (95% CI, 0.90–0.96), indicating strong inter-rater reliability, as was observed for the items on the checklist (Supplementary Table 2). The Spearman correlation coefficient was 0.79 (95% CI, 0.67–0.87) between the total score and the global score, indicative of strong correlation. The standardized Cronbach’s alpha coefficient was 0.75, suggesting good internal reliability.

**Table 2:** The characteristics of the participants and emergency residents

Variables	Trainees (*n* = 50)	Emergency residents (*n* = 12)	p-Value
Age			< 0.0001
≦25years	42 (84%)	0	
26–30 years	8 (16%)	12 (100%)	
Sex			0.578
Females	10 (20%)	2 (17%)	
Males	40 (80%)	10 (83%)	
Ultrasound experience			< 0.0001
≦10 cases	47 (94%)	0	
11–20 cases	2 (4%)	0	
≧ 20 cases	1 (2%)	12 (100%)	
Ultrasound performance^*^			
Ultrasound-guided localization	5 (3–5)	5 (5)	0.004
Visualization of needle	5 (3–5)	5 (5)	0.011
Fluid aspiration	5 (3–5)	5 (5)	0.012
Needle steadiness during aspiration of fluid	5 (5)	5 (5)	0.223
Total score	18 (15–20)	20 (20)	0.003
Global score	5 (4–5)	5 (5)	0.009
Acceptable performance (rating ≧ 3)	50 (100%)	12 (100%)	1.000

*presented with median and interquartile ranges

While the performance of emergency residents surpassed that of trainees, all trainees demonstrated acceptable performance (global rating of ≧ 3). Trainees exhibited less familiarity with US-guided localization, visualization of the needle, and fluid aspiration (Table 2). No significant differences were found in the performance between PGYs and UGYs (Supplementary Table 3).

### Phantom

There were no significant differences observed in terms of image stimulation, puncture texture, needle visualization, drainage simulation, and endurance of the phantom between emergency residents and trainees. However, it is noteworthy that the residents rated puncture texture and draining fluid as “neutral (Table 3). Trainees reported increased confidence in paracentesis after using the phantom, compared with their pre-curriculum survey [4 (3–5) vs. 1 (1), p < 0.0001].

**Table 3:** The feedback to the phantom

Variables*^†^	Trainees (*n* = 50)	Emergency residents (*n* = 12)	p-Value
The sonographic image mimics human tissues	4 (3–4)	4 (3–4)	0.388
The puncture texture mimics human skin and the subcutaneous area	4 (3–4)	3 (3–4)	0.353
The needle can be visualized during the puncture	4 (4–5)	4 (3–5)	0.601
Draining fluid is realistic	4 (3–5)	3 (3–4)	0.072
The phantom is durable	4 (3–5)	4 (3–5)	0.523

*presented with median and interquartile ranges

^†^rated as 1 = strongly disagree; 2 = disagree; 3 = neutral; 4 = agree; 5 = strongly agree

The US phantom could be utilized at least 30 times for practicing paracentesis within one curriculum. The cost of the handmade phantom with a container was approximately $16. Without the container, the cost was reduced to approximately $6.

## Discussion

Commercial US phantoms for paracentesis remain extremely expensive rendering them inaccessible for many training centers. Inexpensive, do-it-yourself phantoms play a crucial role in paracentesis training. In this study, we presented a low-cost, and easily reproducible phantom with echogenicity similar to human tissue and proved its feasibility. Utilizing the phantom facilitates the acquisition of paracentesis skills among novices, enhancing their US abilities and boosting their confidence. While novices demonstrated acceptable performance in paracentesis, it still lags behind that of experienced residents.

Apart from their higher cost, commercial phantoms may degrade with repeated use, requiring an additional fee for fixation. These phantoms typically incorporate polymers, resulting in an excessively firm texture. In contrast, our agar phantom, while having a semi-firm texture that may not perfectly replicate human skin, received a median rating ranging from 3 to 4 from experienced emergency residents in terms of feedback, encompassing image stimulation, puncture texture, needle visualization, and drainage simulation.

Reviewing the literature, some examples of inexpensive, handmade paracentesis phantoms were reported. Wilson et al. documented a gelatin phantom [18], and Kei et al. employed a water jug covered with pork belly [20]. Mesquita et al. used multiple gloves filled with various colors to simulate ascites and abdominal organs, elucidating students’ perceptions of the simulator [19]. In our study, we contribute additional evidence supporting the viability of a handmade phantom, reporting on the performance and feedback of novices in comparison to experienced residents.

Moreover, our phantom exhibited variability and flexibility. For instance, the fluid within the phantom could be altered to appear red or include debris content (such as adding talc), replicating hemoperitoneum or pus, respectively. Additionally, the ratio of fluid to ropes could be adjusted to simulate either a small or a large amount of ascites, depending on the desired training difficulty.

Lower-fidelity modalities are designed to concentrate on a specific learning task and skill acquisition, making them suitable for early learners or novices. In contrast, higher-fidelity simulations are employed for complex tasks, providing cognitive stimuli [1]. Our handmade phantom is a tool with lower fidelity in external appearance but exhibits high fidelity in ultrasound appearance, making it well-suited for paracentesis training, with novices demonstrating proficiency after completing the curriculum. It is important to note that the long-term impact on skill retention and the translation of acquired skills to proficiency in clinical settings remains unknown.

Gelatin is frequently employed as the primary substrate for homemade phantoms in ultrasound training [12, 18, 22]. However, gelatin necessitates refrigeration to solidify the model. In contrast, agar serves as a vegan-friendly alternative that can set the model without the need for refrigeration. Agar is capable of producing an ultrasound image that closely mimics real tissue and is durable enough to withstand high-volume training [23]. In this study, we opted for agar as the substrate, and the resulting echogenicity was deemed acceptable.

The assessment form was developed through expert consensus to ensure content validity. Our results also demonstrated good internal and interrater reliability of the assessment form. Research indicates that global rating scales effectively capture various proficiency levels compared to checklists and are user-friendly for examiners [24]. In this study, the global rating score was utilized to evaluate performance in conjunction with the items on the assessment form.

The main limitation of this study was the inclusion of trainees and emergency residents from a single institution, who voluntarily participated and exhibited high motivation, potentially introducing selection bias. Therefore, caution should be exercised when generalizing these results. Secondly, the study involved a substantial amount of labor and time, approximately 30–60 min for agar preparation and an additional 30 min for phantom assembly, which could limit its feasibility due to time constraints. Third, there may be a potential issue with the image quality of the phantom, as a small amount of air might have been introduced into the balloon during fluid aspiration. However, both trainees and residents reported acceptable image quality. Fourth, while the trainees had prior experience in blood drawing and needle catheterization through routine medical training, feedback concerning human tissue and draining sensation should be interpreted cautiously. Notably, residents with real-world experience rated “neutral” on aspects such as “puncture texture mimics human skin and subcutaneous area” and “draining fluid is realistic.” Lastly, the focus of this study was on evaluating the feasibility of the phantom. Factors such as skill retention and the clinical performance of trainees in real-world scenarios were not investigated. Additionally, the learning effect of using handmade phantoms was not compared with that of using commercial phantoms due to the latter’s high cost. These aspects should be addressed in future studies.

## Conclusion

The paracentesis phantom proves to be a practical and cost-effective training tool. It facilitates the acquisition of paracentesis skills among novices, enhancing their US abilities and boosting their confidence. Nevertheless, further investigation is needed to assess its skill retention and long-term impact on clinical performance in real patients.

### Electronic supplementary material

Below is the link to the electronic supplementary material.

Supplementary Material 1

Supplementary Material 2

Supplementary Material 3

Supplementary Material 4

## Data Availability

All data analyzed during this study are included in this published article.

## Abbreviations

Ultrasound: US
National Taiwan University Hospital: NTUH
Postgraduate-year: PGY
Undergraduate-year: UGY
Interquartile range: IQR
Intraclass correlation coefficient: ICC
Confidence interval: CI
