# Supplementary material for: A feasibility study of a handmade ultrasound-guided phantom for paracentesis
Source: BMC Med Educ. 2024 Mar 29;24:351. doi: 10.1186/s12909-024-05339-9 (PMC10981280; doi:10.1186/s12909-024-05339-9)
Supplement: Supplementary file 2 — Supplementary Material 2 [file 12909_2024_5339_MOESM2_ESM.docx]

Supplementary Table 1. The survey of feedback.

| Question | 1 | 2 | 3 | 4 | 5 |
| --- | --- | --- | --- | --- | --- |
| 1. The sonographic image mimics human tissues | strongly disagree | disagree | neutral | agree | strongly agree |
| 1. The puncture texture mimics human skin and the subcutaneous area | strongly disagree | disagree | neutral | agree | strongly agree |
| 1. The needle can be visualized during the puncture | strongly disagree | disagree | neutral | agree | strongly agree |
| 1. Draining fluid is realistic | strongly disagree | disagree | neutral | agree | strongly agree |
| 1. The phantom is durable | strongly disagree | disagree | neutral | agree | strongly agree |
