# Supplementary material for: A feasibility study of a handmade ultrasound-guided phantom for paracentesis
Source: BMC Med Educ. 2024 Mar 29;24:351. doi: 10.1186/s12909-024-05339-9 (PMC10981280; doi:10.1186/s12909-024-05339-9)
Supplement: Supplementary file 3 — Supplementary Material 3 [file 12909_2024_5339_MOESM3_ESM.docx]

Supplementary Table 2. The interrater reliability using the intraclass correlation coefficient.

| Assessment | Score^*^ | Intraclass correlation coefficient (95% CI^†^) |
| --- | --- | --- |
| Ultrasound-guided localization | 5 (3-5) | 0.96 (0.94-0.98) |
| Visualization of needle | 5 (3-5) | 0.92 (0.88-0.95) |
| Fluid aspiration | 5 (4-5) | 0.84 (0.75-0.90) |
| Needle steadiness during aspiration of fluid | 5 (5) | 0.88 (0.82-0.93) |
| Total score | 20 (16-20) | 0.98 (0.98-0.99) |
| Global score | 5 (4-5) | 0.94 (0.90-0.96) |

^*^presented with median and interquartile ranges.

^†^CI=confidence interval.
