# Supplementary material for: A feasibility study of a handmade ultrasound-guided phantom for paracentesis
Source: BMC Med Educ. 2024 Mar 29;24:351. doi: 10.1186/s12909-024-05339-9 (PMC10981280; doi:10.1186/s12909-024-05339-9)
Supplement: Supplementary file 4 — Supplementary Material 4 [file 12909_2024_5339_MOESM4_ESM.docx]

Supplementary Table 3. The ultrasound performance and feedback to the phantom the post-graduate-year (PGY) residents and undergraduate-year (UGY) students.

| Variables | PGY (n=22) | | UGY (n=28) | | p-Value | | |
| --- | --- | --- | --- | --- | --- | --- | --- |
| Ultrasound performance^*^ | |  | |  | |  | |
| Ultrasound-guided localization | | 5 (3-5) | | 5 (3-5) | | 0.556 | |
| Visualization of needle | | 5 (3-5) | | 5 (3-5) | | 0.739 | |
| Fluid aspiration | | 5 (3-5) | | 5 (3-5) | | 0.890 | |
| Needle steadiness during aspiration of fluid | | 5 (5) | | 5 (5) | | 0.273 | |
| Total score | | 18 (16-20) | | 18 (15-20) | | 0.896 | |
| Global score^*^ | | 5 (4-5) | | 5 (4-5) | | 0.825 | |
| Feedback^*^ | |  | |  | |  | |
| Image stimulation | | 4 (3-4) | | 4 (3-4) | | 0.896 | |
| Puncture texture | | 4 (3-4) | | 4 (3-4) | | 0.469 | |
| Needle visualization | | 4 (4-5) | | 4 (4-5) | | 0.296 | |
| Drainage simulation | | 4 (3-5) | | 4 (3.5-5) | | 0.250 | |
| Endurance | | 4 (3-5) | | 4 (3-5) | | 0.931 | |
| *presented with median and interquartile ranges. | | | | | | | |
